# Supplementary material for: Multiple pkd and piezo gene family members are required for atrioventricular valve formation
Source: Nat Commun. 2023 Jan 13;14:214. doi: 10.1038/s41467-023-35843-3 (PMC9839778; doi:10.1038/s41467-023-35843-3)
Supplement: Supplementary file 1 — Supplementary Information [file 41467_2023_35843_MOESM1_ESM.pdf]

SUPPLEMENTARY INFORMATION

Multiple *pkd* and *piezo* gene family members are required for atrioventricular valve formation

Thomas Juan[1,2,3,*], Agatha Ribeiro Da Silva[1,2,†], Barbara Cardoso[1,2,†], SoEun Lim[1,2,], Violette Charteau[1,2,4], Didier Y.R. Stainier[1, 2,3,*]

1. Max Planck Institute for Heart and Lung Research, Department of Developmental Genetics, Bad Nauheim, Germany.
2. German Centre for Cardiovascular Research (DZHK), Partner Site Rhine-Main, Bad Nauheim, Germany.
3. Cardio-Pulmonary Institute (CPI), Bad Nauheim, Germany.
4. Current address: Institute for Molecules and Materials (IMM), Department of Biomolecular Chemistry, Radboud University, Nijmegen, The Netherlands.

† These authors contributed equally to this work.
* Correspondence to: Thomas Juan - thomas.juan@mpi-bn.mpg.de or to Didier Y. R. Stainier - didier.stainier@mpi-bn.mpg.de
Max Planck Institute for Heart and Lung Research, Ludwigstr. 43, 61231 Bad Nauheim, Germany

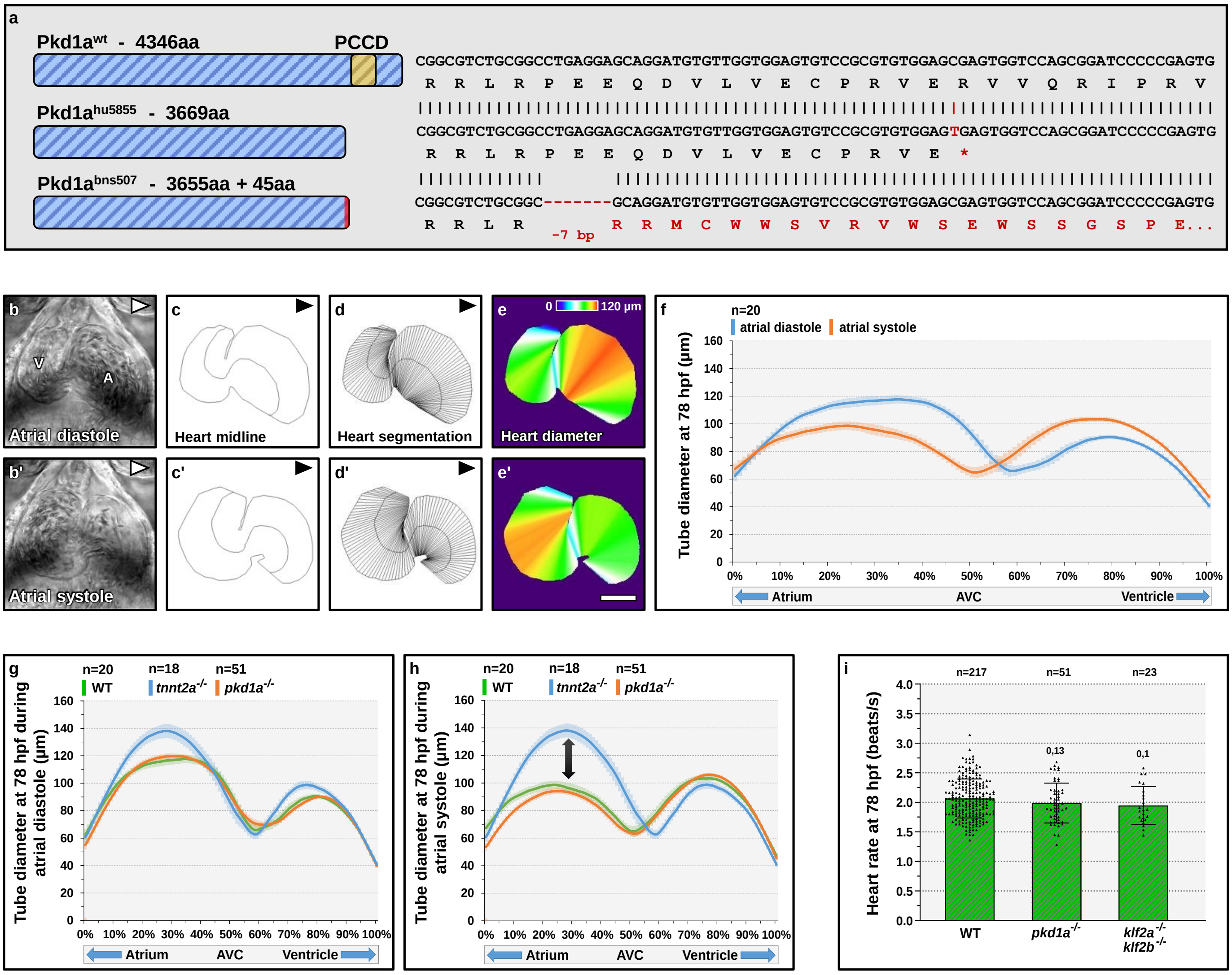

Fig. S1 Wild-type-like cardiac chamber size in 78 hpf *pkd1a* mutants. a Schematics of Pkd1a (wild-type and predicted mutants) showing the disruption of the conserved PCCD. b-e' Diameter quantification method along the heart tube during atrial diastole (b-e) and systole (b'-e'); brightfield images of hearts (b,b') are segmented along the heart midline (c-d') and displayed using a thermal lookup table (e,e'). f Diameter along the heart tube of 78 hpf wild-type larvae during atrial diastole and systole. g-h Diameter along the heart tube of 78 hpf wild-type, *tnnt2a* mutant, and *pkd1a* mutant larvae during atrial diastole (g) and systole (h)); arrow points to the decreased heart diameter during atrial systole. i Heart rate of 78 hpf wild-type, *pkd1a* mutant, and *klf2a; klf2b* double mutant larvae. The center of the error bar represents the mean in i. Error bars indicate S.E.M. in f, g, h; s.d. in i. P-values were calculated using a two-sided Student's t-test in i and are relative to wild type. Scale bar: 50 µm. Source data are provided as a Source Data file.

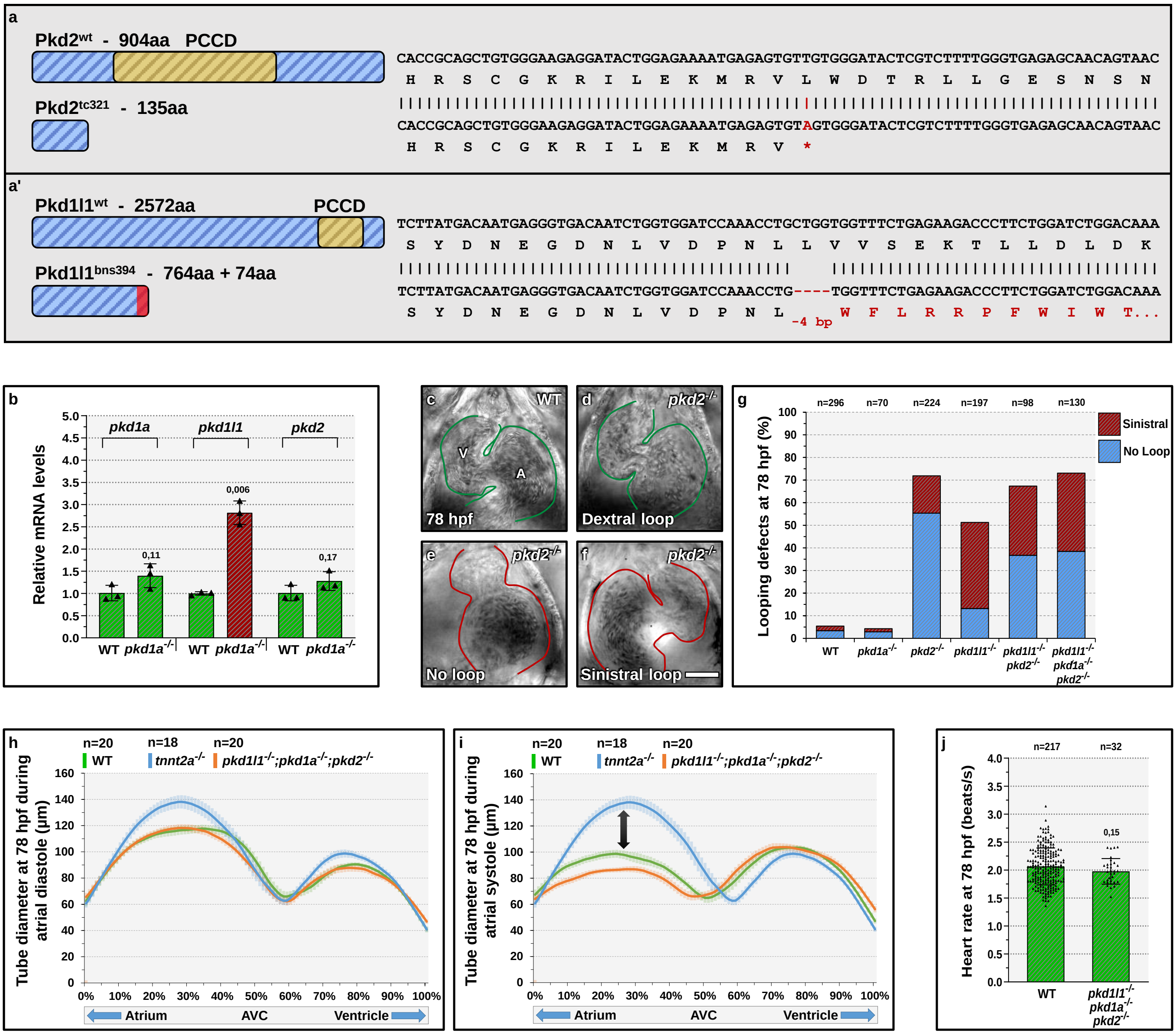

Fig. S2 *pkd2 and pkd1l1* mutants display heart looping defects. a-a' Schematics of Pkd2 (a) and Pkd1l1 (a') (wild-type and predicted mutants) showing the disruption of the conserved PCCD. b Relative mRNA levels of *pkd1a*, *pkd1l1*, and *pkd2* in 78 hpf wild types and *pkd1a* mutants; n= 3 biologically independent samples; Ct values are listed in Supplementary Data 1. c-f Brightfield images of 78 hpf wild-type (c) and *pkd2* mutant hearts which display normal (d) or defective looping (e-f); one time point of a spinning disc movie at atrial diastole; green and red lines outline the heart. g Heart looping defects of 78 hpf wild-types and *pkd* mutants. h-i Diameter along the heart tube of 78 hpf wild-type, *tnnt2a* mutant, and *pkd* triple mutant larvae during atrial diastole (h) and systole (i); arrow points to the decreased heart diameter during atrial systole. j Heart rate of 78 hpf wild-type and *pkd* triple mutant larvae. Hearts imaged in ventral view, anterior to the top, ventricle (V) on the left and atrium (A) on the right. The center of the error bar represents the mean in b, j. Error bars indicate S.E.M. in h, i; s.d. in b, j. P-values were calculated using a two-sided Student's t-test in b, j and are relative to wild type. Scale bar: 50 µm. Source data are provided as a Source Data file.

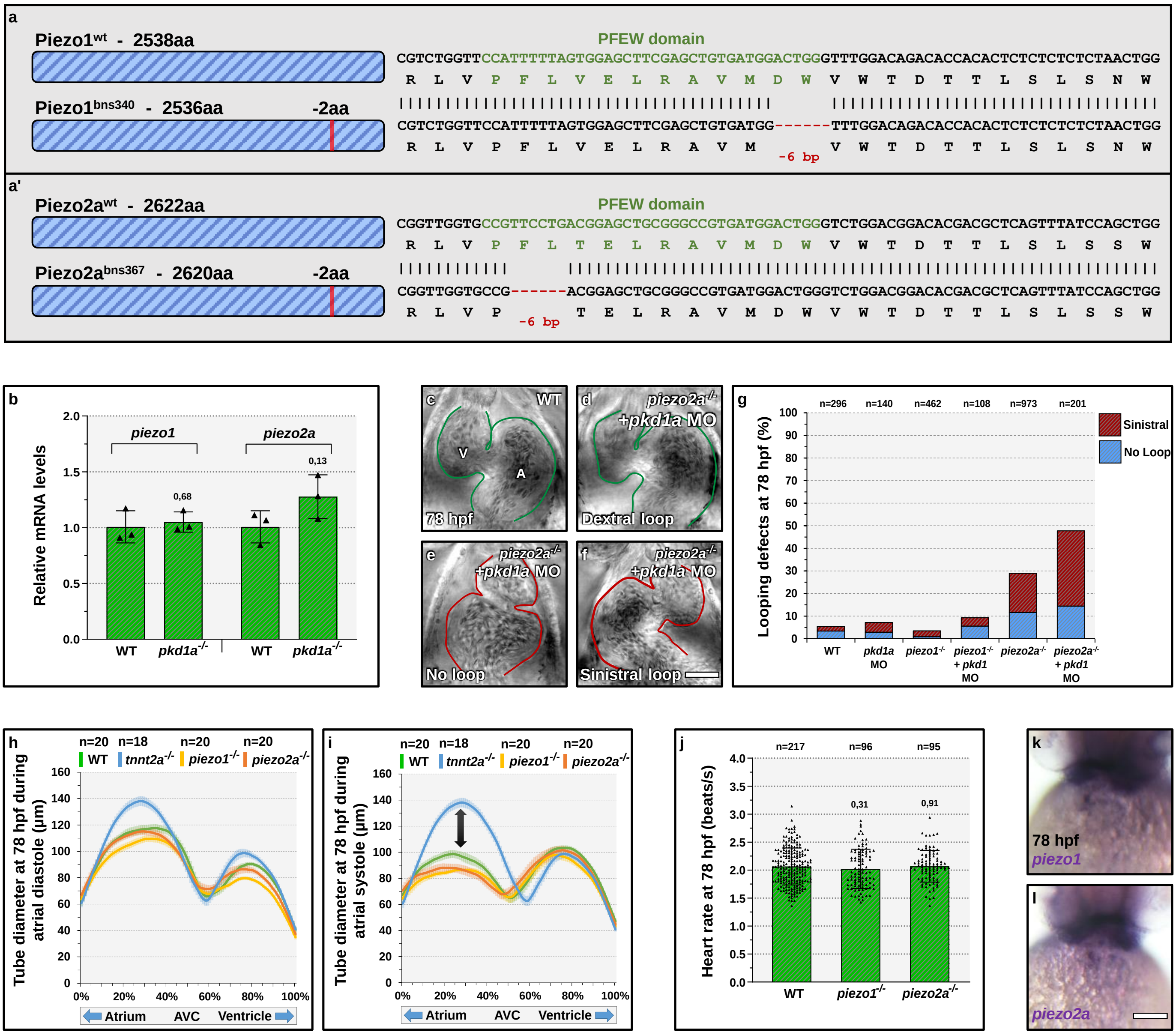

Fig. S3 *piezo2a* mutants display heart looping defects. a-a' Schematics of Piezo1 (a) and Piezo2 (a') (wild-type and predicted mutants) showing the disruption of the conserved PFEW domain. b Relative mRNA levels of *piezo1* and *piezo2a* in 78 hpf wild types and *pkd1a* mutants; n= 3 biologically independent samples; Ct values are listed in Supplementary Data 1. c-f Brightfield images of a 78 hpf wild type (c) and 78 hpf *piezo2a* mutants injected at the one-cell stage with a *pkd1a* morpholino which display normal (d) or defective (e-f) cardiac looping; one time point of a spinning disc movie at atrial diastole; green and red lines outline the heart. g Heart looping defects of 78 hpf wild-type, *piezo1* mutant, and *piezo2a* mutant larvae, non-injected or injected at the one-cell stage with a *pkd1a* morpholino. h-i Diameter along the heart tube of 78 hpf wild-type, *tnnt2a* mutant, *piezo1* mutant, and *piezo2a* mutant larvae during atrial diastole (h) and systole (i); arrow point to the decreased heart diameter during atrial systole state. j Heart rate of 78 hpf wild-type, *piezo1* mutant, *piezo2a* mutant, and *piezo2a; pkd1a* double mutant larvae. k-l *In situ* hybridization at 78 hpf for *piezo1* (k) and *piezo2a* (l) expression. Hearts imaged in ventral view, anterior to the top, ventricle (V) on the left and atrium (A) on the right. The center of the error bar represents the mean in b, j. Error bars indicate S.E.M. in h, i; s.d. in b, j. P-values were calculated using a two-sided Student's t-test in b, j and are relative to wild type. Scale bars: 100 µm in k,l; 50 µm in c-f. Source data are provided as a Source Data file.

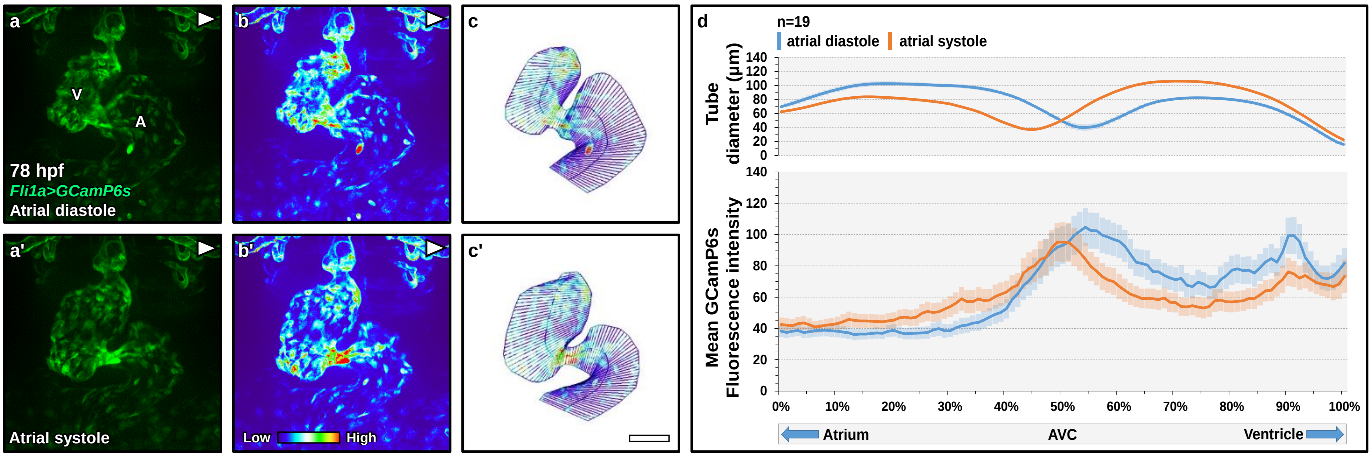

Fig. S4 Elevated calcium levels in the atrioventricular canal at an early larval stage. a-c' Confocal imaging and quantification method of 78hpf *fli1a:Gal4FF; UAS:GCamP6s* hearts during atrial diastole (a) and systole (a'), displayed using a thermal lookup table (b,b') and segmented along the cardiac midline using the GCamP6s fluorescence signal (c,c'); maximum projection of 4D-assembled spinning disc movies. d Diameter and endocardial calcium levels along the contracting heart tube in 78 hpf wild-type larvae during atrial diastole and systole. Hearts imaged in ventral views, anterior to the top, ventricle (V) on the left and atrium (A) on the right. Error bars indicate S.E.M. Scale bar: 50 µm. Source data are provided as a Source Data file.

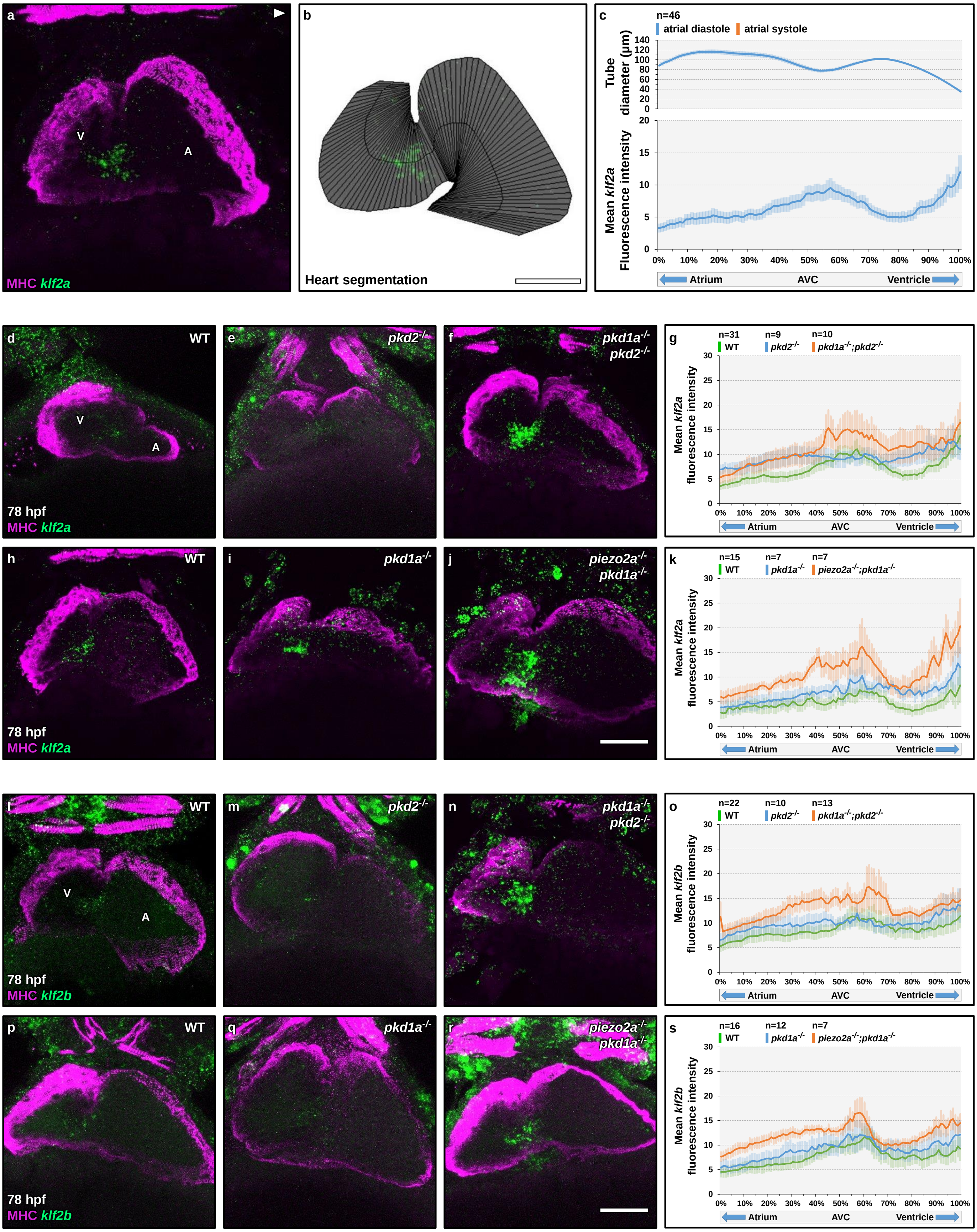

Fig. S5 Transcriptional modulation in the atrioventricular canal by Pkd and Piezo members. a-b Confocal projection and quantification method of a 78 hpf wild-type heart stained for MHC and *klf2a* (a), segmented along the cardiac midline using MHC staining to analyze *klf2a* mean fluorescence intensity (b). c *klf2a* mean fluorescence intensity along the heart tube in 78 hpf wild-type larvae. d-f, h-j, l-n, p-r Confocal projections of 78 hpf wild-type (d,h,l,p), *pkd2* mutant (e,m), *pkd1a* mutant (i,q), *pkd1a; pkd2* double mutant (f,n), and *piezo2a; pkd1a* double mutant (j,r) hearts stained for MHC, and *klf2a* (d-f, h-j), or *klf2b* (l-n, p-r) expression. g, k, o, s *klf2a* (g,k) and *klf2b* (o,s) mean fluorescence intensity along the heart tube in 78 hpf wild-type, *pkd2* mutant, *pkd1a* mutant, *pkd1a; pkd2* double mutant, and *piezo2a; pkd1a* double mutant larvae. Hearts imaged in ventral view, anterior to the top, ventricle (V) on the left and atrium (A) on the right. Error bars indicate S.E.M. in c, g, k, o, s. Scale bars: 50 µm. Source data are provided as a Source Data file.

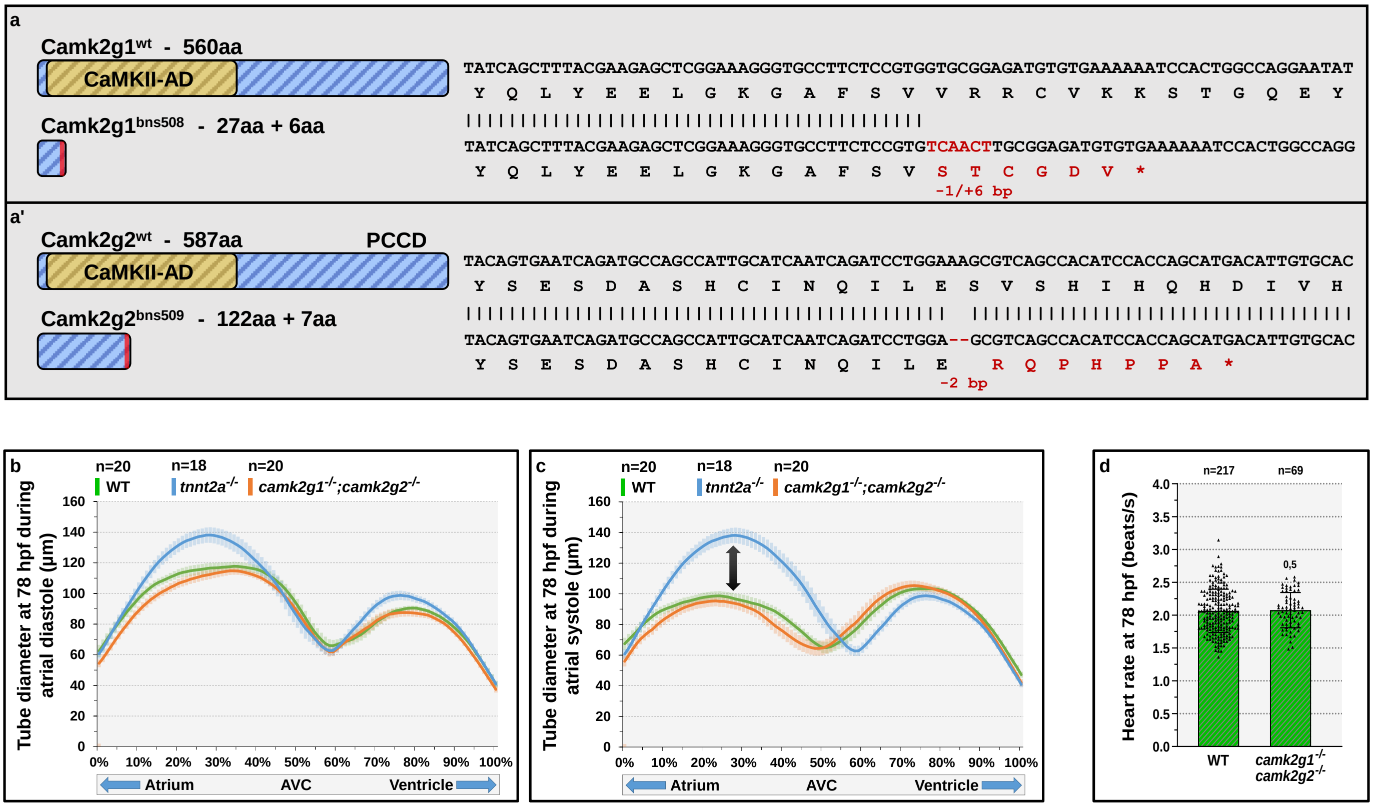

Fig. S6 Wild-type-like cardiac chamber size in 78 hpf *camk2g* mutants. a-a' Schematics of Camk2g1 (a) and Camk2g2 (a') (wild-type and predicted mutants) showing the disruption of the conserved CamKII-AD. b-c Diameter along the heart tube in 78 hpf wild-type, *tnnt2a* mutant, and *camk2g1; camk2g2* double mutant larvae during atrial diastole (b) and systole (c); arrow points to the decreased heart diameter during atrial systole. d Heart rate of 78 hpf wild-type, *camk2g1* mutant, *camk2g2* mutant, and *camk2g1; camk2g2* double mutant larvae. The center of the error bar represents the mean in d. Error bars indicate S.E.M. in b,c; s.d. in d. P-values are calculated using a two-sided Student's t-test in d and are relative to wild type. Source data are provided as a Source Data file.
