# Supplementary material for: Multiple pkd and piezo gene family members are required for atrioventricular valve formation
Source: Nat Commun. 2023 Jan 13;14:214. doi: 10.1038/s41467-023-35843-3 (PMC9839778; doi:10.1038/s41467-023-35843-3)
Supplement: Supplementary file 3 — Description of Additional Supplementary Files [file 41467_2023_35843_MOESM3_ESM.pdf]

Description of Additional Supplementary Files

File Name: Supplementary Data 1
Description: CRISPR sites, primers, and Ct values.

File Name: Supplementary Movie 1
Description: Brightfield live imaging (4.13 msec/frame) of a 54 hpf wild-type heart. The movie depicts the AV flow profile (anterograde, no-flow, and retrograde) during 3 cardiac cycles. Scale bar: 50 µm.

File Name: Supplementary Movie 2
Description: Brightfield live imaging (4.13 msec/frame) of a 78 hpf wild-type heart. The movie depicts the AV flow profile (anterograde, no-flow, and retrograde) during 3 cardiac cycles. Scale bar: 50 µm.

File Name: Supplementary Movie 3
Description: Brightfield live imaging (4.13 msec/frame) of a 102 hpf wild-type heart. The movie depicts the AV flow profile (anterograde, no-flow, and retrograde) during 3 cardiac cycles. Scale bar: 50 µm.

File Name: Supplementary Movie 4
Description: Brightfield live imaging (4.13 msec/frame) of a 78 hpf *pkd1a* mutant heart. The movie depicts the AV flow profile (anterograde, no-flow, and retrograde) during 3 cardiac cycles. Scale bar: 50 µm.

File Name: Supplementary Movie 5
Description: Brightfield live imaging (4.13 msec/frame) of a 78 hpf *pkd1l1*; *pkd1a*; *pkd2* triple mutant heart. The movie depicts the AV flow profile (anterograde, no-flow, and retrograde) during 3 cardiac cycles. Scale bar: 50 µm.

File Name: Supplementary Movie 6
Description: Calcium live imaging (30 msec/frame) of a 78 hpf wild-type heart using the *fli1a:Gal4FF; UAS:GCamP6s* endothelial calcium sensor line during 3 cardiac cycles. Maximal projection of a 4D-assembled spinning disc movie. Scale bar: 50 µm.

File Name: Supplementary Movie 7
Description: Calcium live imaging (30 msec/frame) of a 78 hpf *pkd1l1*; *pkd1a*; *pkd2* triple mutant heart using the *fli1a:Gal4FF; UAS:GCamP6s* endothelial calcium sensor line during 3 cardiac cycles. Maximal projection of a 4D-assembled spinning disc movie. Scale bar: 50 µm.

File Name: Supplementary Movie 8
Description: Calcium live imaging (30 msec/frame) of a 78 hpf *tnnt2a* morphant heart using the *fli1a:Gal4FF; UAS:GCamP6s* endothelial calcium sensor line during 3 cardiac cycles. Maximal projection of a 4D-assembled spinning disc movie. Scale bar: 50 µm.
