# Supplementary material for: Multiple pkd and piezo gene family members are required for atrioventricular valve formation
Source: Nat Commun. 2023 Jan 13;14:214. doi: 10.1038/s41467-023-35843-3 (PMC9839778; doi:10.1038/s41467-023-35843-3)
Supplement: Supplementary file 13 — Reporting Summary [file 41467_2023_35843_MOESM13_ESM.pdf]

nature portfolio

Corresponding author(s): Didier Y.R. Stainier
Thomas Juan

Last updated by author(s): Dec 20, 2022

Reporting Summary

Nature Portfolio wishes to improve the reproducibility of the work that we publish. This form provides structure for consistency and transparency in reporting. For further information on Nature Portfolio policies, see our Editorial Policies and the Editorial Policy Checklist.

Statistics

For all statistical analyses, confirm that the following items are present in the figure legend, table legend, main text, or Methods section.

n/a	Confirmed	
☐	☒	The exact sample size (*n*) for each experimental group/condition, given as a discrete number and unit of measurement
☐	☒	A statement on whether measurements were taken from distinct samples or whether the same sample was measured repeatedly
☐	☒	The statistical test(s) used AND whether they are one- or two-sided<br>*Only common tests should be described solely by name; describe more complex techniques in the Methods section.*
☒	☐	A description of all covariates tested
☒	☐	A description of any assumptions or corrections, such as tests of normality and adjustment for multiple comparisons
☐	☒	A full description of the statistical parameters including central tendency (e.g. means) or other basic estimates (e.g. regression coefficient) AND variation (e.g. standard deviation) or associated estimates of uncertainty (e.g. confidence intervals)
☐	☒	For null hypothesis testing, the test statistic (e.g. *F*, *t*, *r*) with confidence intervals, effect sizes, degrees of freedom and *P* value noted<br>*Give P values as exact values whenever suitable.*
☒	☐	For Bayesian analysis, information on the choice of priors and Markov chain Monte Carlo settings
☒	☐	For hierarchical and complex designs, identification of the appropriate level for tests and full reporting of outcomes
☒	☐	Estimates of effect sizes (e.g. Cohen's *d*, Pearson's *r*), indicating how they were calculated

Our web collection on statistics for biologists contains articles on many of the points above.

Software and code

Policy information about availability of computer code

Data collection	Microscopy images were acquired with NIS-Elements 4.30, ZEN 2011, ZEN 2.5 Blue, and ZEN. 3.5 Blue. RT-qPCR data was acquired with Bio-Rad CFX Manager 3.1.
Data analysis	AV flow profile analysis, valve elongation quantification, and heart segmentation were performed using Fiji (ImageJ 1.53q). Sequence analysis was performed using ApE (v2.0.54). Data were processed with GraphPad Prism 9 and Microsoft excel 2016.

For manuscripts utilizing custom algorithms or software that are central to the research but not yet described in published literature, software must be made available to editors and reviewers. We strongly encourage code deposition in a community repository (e.g. GitHub). See the Nature Portfolio guidelines for submitting code & software for further information.

Data

Policy information about availability of data

All manuscripts must include a data availability statement. This statement should provide the following information, where applicable:

- Accession codes, unique identifiers, or web links for publicly available datasets
- A description of any restrictions on data availability
- For clinical datasets or third party data, please ensure that the statement adheres to our policy

Materials and raw images that support the findings of this study are available upon request to the corresponding authors. Raw data are provided as a Source Data file.

Human research participants

Policy information about studies involving human research participants and Sex and Gender in Research.

Reporting on sex and gender	N/A
Population characteristics	N/A
Recruitment	N/A
Ethics oversight	N/A

Note that full information on the approval of the study protocol must also be provided in the manuscript.

Field-specific reporting

Please select the one below that is the best fit for your research. If you are not sure, read the appropriate sections before making your selection.

☒ Life sciences ☐ Behavioural & social sciences ☐ Ecological, evolutionary & environmental sciences

For a reference copy of the document with all sections, see nature.com/documents/nr-reporting-summary-flat.pdf

Life sciences study design

All studies must disclose on these points even when the disclosure is negative.

Sample size	Sample sizes were chosen based on accepted standards in the field and previously published literature (such as PMID: 34648325, 34422841, or 32070236). For each measurement condition, >5 embryos/larvae were analyzed. No statistical method was used to predetermine the sample size.
Data exclusions	No data were excluded from analysis.
Replication	All experiments were verified with at least 3 biological replicates or 3 independent experiments.
Randomization	For injection experiments, the embryos were randomly allocated to control or mRNA injected groups by randomly dividing half of a clutch at the 1-cell-stage. For other experiments, the embryos/larvae were selected randomly, genotyped after the experiment for zygotic mutants, and randomized into different groups.
Blinding	Blinding was performed for embryo/larvae collection, and imaging/data analysis before genotyping.

Reporting for specific materials, systems and methods

We require information from authors about some types of materials, experimental systems and methods used in many studies. Here, indicate whether each material, system or method listed is relevant to your study. If you are not sure if a list item applies to your research, read the appropriate section before selecting a response.

Materials & experimental systems

n/a	Involved in the study	
☐	☒	Antibodies
☒	☐	Eukaryotic cell lines
☒	☐	Palaeontology and archaeology
☐	☒	Animals and other organisms
☒	☐	Clinical data
☒	☐	Dual use research of concern

Methods

n/a	Involved in the study	
☒	☐	ChIP-seq
☒	☐	Flow cytometry
☒	☐	MRI-based neuroimaging

Antibodies

Antibodies used	The following antibody were used: anti-MHC (MF20) (Invitrogen 14-6503-82), anti-Alcama (ZN-8, DSHB), and anti-Fli1 (abcam ab133485), anti-DIG-POD (Roche 11207733910)
Validation	anti-MHC (MF20), https://www.thermofisher.com/antibody/product/Myosin-4-Antibody-clone-MF20-Monoclonal/14-6503-82

Validation	Manufacturer: "This Antibody was verified by Cell treatment to ensure that the antibody binds to the antigen stated."<br><br>anti-Alcama, https://dshb.biology.uiowa.edu/ZN-8<br>Manufacturer: "Works on zebrafish embryos/larva and adults."<br><br>anti-Fli1, https://www.abcam.com/fli1-antibody-epr4646-ab133485.html<br>Reference: PMID 32850848<br><br>anti-DIG-POD, https://www.sigmaaldrich.com/DE/en/product/roche/11207733910<br>Manufacturer: "The polyclonal antibody from sheep is specific to digoxigenin and digoxin"

Animals and other research organisms

Policy information about studies involving animals; ARRIVE guidelines recommended for reporting animal research, and Sex and Gender in Research

Laboratory animals	We used zebrafish (Danio rerio) embryos (54 hpf) and larvae (78 and 102 hpf). The following strains were used in this study: AB, klf2a bns11 (Rasouli et al. 2018), klf2b bns12 (Rasouli et al. 2018), pkd1a hu5855 (Coxam et al. 2014) pkd2 tc321 (Schottenfeld et al. 2017), tnnt2a gbt-R14 (Clarck et al. 20111), Tg(fli1a:Gal4FF)ubs4 (Herwig et al. 2011), Tg(UAS:GCamP6s)nkUAShspzGCaMP6s13a (Muto et al. 2017), and TgBAC(nfatc1:Gal4)mu286 (Pestel et al. 2016).
Wild animals	No wild animals were used in the study.
Reporting on sex	Our study is performed on early developmental stages, before sex differentiation.
Field-collected samples	No field collected samples were used in the study.
Ethics oversight	All procedures performed on animals conform to the guidelines from Directive 2010/63/EU of the European Parliament on the protection of animals used for scientific purposes and were approved by the Animal Protection Committee (Tierschutzkommission) of the Regierungspräsidium Darmstadt (reference: B2/1218).

Note that full information on the approval of the study protocol must also be provided in the manuscript.
